# HIVseqDB: a portable resource for NGS and sample metadata integration for HIV-1 drug resistance analysis

**DOI:** 10.1093/bioadv/vbae008

**Published:** 2024-01-14

**Authors:** Alfred Ssekagiri, Daudi Jjingo, Nicholas Bbosa, Daniel L Bugembe, David P Kateete, I King Jordan, Pontiano Kaleebu, Deogratius Ssemwanga

**Affiliations:** Department of General Virology, Uganda Virus Research Institute, Entebbe 31405, Uganda; Department of Immunology and Molecular Biology, Makerere University, Kampala 10206, Uganda; Department of Computer Science, Makerere University, Kampala 10207, Uganda; African Centre of Excellence in Bioinformatics and Data Intensive Sciences, Makerere University, Kampala 10207, Uganda; Medical Research Council (MRC)/Uganda Virus Research Institute (UVRI) and London School of Hygiene and Tropical Medicine (LSHTM) Uganda Research Unit, Entebbe 31405, Uganda; Medical Research Council (MRC)/Uganda Virus Research Institute (UVRI) and London School of Hygiene and Tropical Medicine (LSHTM) Uganda Research Unit, Entebbe 31405, Uganda; Department of Immunology and Molecular Biology, Makerere University, Kampala 10206, Uganda; School of Biological Sciences, Georgia Institute of Technology, Atlanta, GA 30332, United States; Department of General Virology, Uganda Virus Research Institute, Entebbe 31405, Uganda; Medical Research Council (MRC)/Uganda Virus Research Institute (UVRI) and London School of Hygiene and Tropical Medicine (LSHTM) Uganda Research Unit, Entebbe 31405, Uganda; Department of General Virology, Uganda Virus Research Institute, Entebbe 31405, Uganda; Medical Research Council (MRC)/Uganda Virus Research Institute (UVRI) and London School of Hygiene and Tropical Medicine (LSHTM) Uganda Research Unit, Entebbe 31405, Uganda

## Abstract

**Summary:**

Human immunodeficiency virus (HIV) remains a public health threat, with drug resistance being a major concern in HIV treatment. Next-generation sequencing (NGS) is a powerful tool for identifying low-abundance drug resistance mutations (LA-DRMs) that conventional Sanger sequencing cannot reliably detect. To fully understand the significance of LA-DRMs, it is necessary to integrate NGS data with clinical and demographic data. However, freely available tools for NGS-based HIV-1 drug resistance analysis do not integrate these data. This poses a challenge in interpretation of the impact of LA-DRMs, mainly for resource-limited settings due to the shortage of bioinformatics expertise. To address this challenge, we present HIVseqDB, a portable, secure, and user-friendly resource for integrating NGS data with associated clinical and demographic data for analysis of HIV drug resistance. HIVseqDB currently supports uploading of NGS data and associated sample data, HIV-1 drug resistance data analysis, browsing of uploaded data, and browsing and visualizing of analysis results. Each function of HIVseqDB corresponds to an individual Django application. This ensures efficient incorporation of additional features with minimal effort. HIVseqDB can be deployed on various computing environments, such as on-premises high-performance computing facilities and cloud-based platforms.

**Availability and implementation:**

HIVseqDB is available at https://github.com/AlfredUg/HIVseqDB. A deployed instance of HIVseqDB is available at https://hivseqdb.org.

## 1 Introduction

Human immunodeficiency virus (HIV) remains a public health threat, with drug resistance being a major concern in HIV treatment (Chimukangara *et al.* 2017). Genotypic testing is used to determine drug resistance, by analyzing the genetic sequence of the virus to identify drug resistance mutations. Next-generation sequencing (NGS) can identify low-abundance drug resistance mutations (LA-DRMs), which could be associated with poor treatment outcomes and cannot reliably be detected by traditional Sanger sequencing (Ávila-Ríos *et al.* 2020). To understand the impact of LA-DRMs in different geographical and clinical settings, it is necessary to integrate clinical and epidemiological data with next-generation sequence data (Mbunkah *et al.* 2020). Furthermore, LA-DRMs have been demonstrated to exhibit a wide range of mutational load (calculated as the product of viral load and mutation frequency), highlighting the importance of incorporating viral load when evaluating the impact of LA-DRMs (Gupta *et al.* 2014). Moreover, the impact of LA-DRMs on the occurrence of treatment failure varies across distinct drug classes and patient populations (Li *et al.* 2021). This underscores the importance of considering demographics and clinical information while assessing the role of LA-DRMs in treatment response.

However, available tools for NGS-based HIV-1 drug resistance analysis, such as HyDRA web and HIVdb-NGS, do not integrate sample data with NGS data.

We present HIVseqDB a portable, secure, and user-friendly resource for storing NGS data with associated clinical and demographic data. The resource can be deployed as a local instance. This ensures data security and proper control of sample metadata provenance, reliable connectivity in settings with unreliable internet coverage, enables exploratory data analysis ahead of sharing data publicly, can be useful for training purposes in resource-limited circumstances (Jjingo *et al.* 2022), and improves analysis turn-around-times. The resource can be adopted by different HIV-1 sequencing laboratories on platforms that are deemed secure enough and accessed by authorized personnel.

## 2 Methods

### 2.1 Back-end architecture

**Figure 1.**
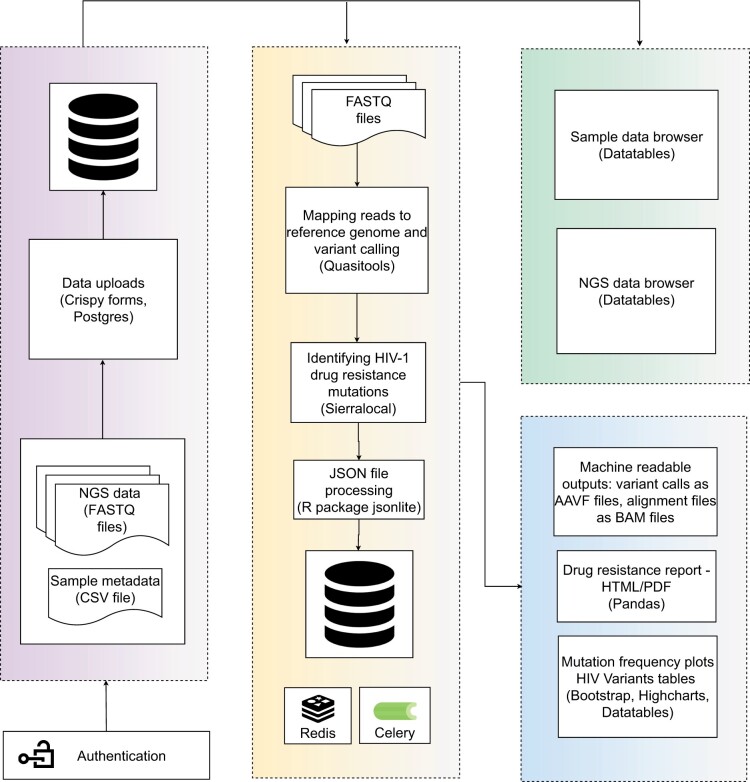
Schematic architecture of HIVseqDB: authenticated users upload data (NGS data and corresponding sample data) to the database. Uploaded data are accessible through the data browser. The analysis layer takes up FASTQ files as input and sends results to database. Machine readable outputs, data tables, and interactive visualizations are generated and are accessible through the analyses browser.

The physical architecture of HIVseqDB is shown in Fig. 1. HIVseqDB is built on Django (https://www.djangoproject.com), an open-source PYTHON web framework, which follows a model-view-template pattern; where, the model manages the data, the view displays the data to the user, and the template defines the structure of the user interface. Currently, the platform consists of four main components; (i) authentication, (ii) data upload and storage of NGS data along with associated sample metadata, (iii) HIV-1 drug resistance analysis, and (iv) browser for data exploration and analyses. These components are implemented as individual applications that communicate with each other to ensure easy customization and the addition of more functionalities with minimal effort. PostgreSQL is used as the main database management system for data storage. Celery (https://docs.celeryq.dev) is used as a task queue to manage background tasks with Redis (https://redis.io/) as the message broker and a caching layer to improve performance. The background tasks primarily include drug resistance analysis, which is handled by quasitools (https://phac-nml.github.io/quasitools/), sierralocal (Ho *et al.* 2019), and R package jsonlite. Nginx (https://www.nginx.com) is used as a reverse proxy server to handle incoming requests and their distribution to appropriate components. These components are all packaged using Docker Compose (https://docs.docker.com), which makes it relatively easy to manage and deploy HIVseqDB across various computing environments.

### 2.2 User interface and access control

The design of HIVseqDB is focused on creating a user-friendly, and engaging experience for users. We used a variety of front-end frameworks and libraries, which include Bootstrap (https://getbootstrap.com) for pre-built user interface components and styles that are responsive and easy to use, Data Tables (https://datatables.net) for easy display of tabular data in a flexible and user-friendly way, Django crispy forms for streamlined form design, and Highcharts (https://www.highcharts.com) for dynamic and interactive visualizations, to make the data more engaging and easier to understand. Users of HIVseqDB are required to login using credentials assigned by the administrator of the resource. Logged in users have access to the data uploads, can create analyses, and browse uploaded data and analyses. Guest users have access to the home page and the documentation of HIVseqDB.

### 2.3 Data management

**Figure 2.**
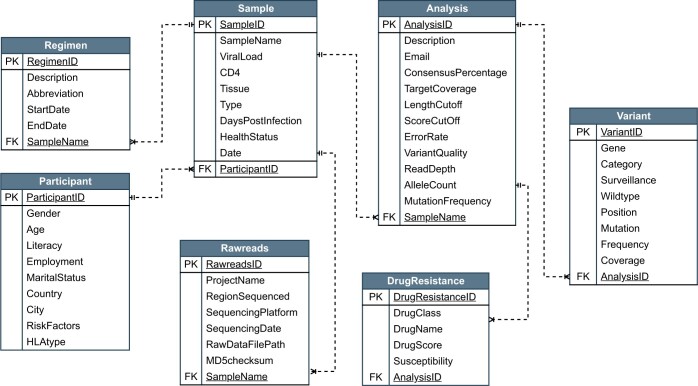
Database schema of HIVseqDB. PK, primary key; FK, foreign key.

To ensure that ethical considerations regarding data usage are meticulously addressed, we emphasize that all data should be anonymized and de-identified before uploading it to HIVseqDB. The database consists of three components; (i) sample data, which includes; sample collection date, sample type, sample tissue; the demographics (age, gender, literacy, employment, country, city, marital status, and risk factors), clinical data (regimen, date of regimen initiation, viral load, CD4 counts, days post infection, and health status), (ii) NGS data, which includes; the project ID, sequencing technology, sequencing platform, sequencing date, sequenced region of HIV-1 genome (e.g. integrase, reverse transcriptase, protease, whole genome, env, pol, and gag), the path to the corresponding FASTQ files, and (iii) drug resistance analysis results (Fig. 2). The data schema of HIVseqDB was developed with reference to similar resources, such as RHIVDB (Tarasova *et al.* 2021), PANGEA database (Abeler-Dörner *et al.* 2019), and Los Alamos HIV sequence database (Foley *et al.* 2018). Data are stored in a PostgreSQL database except for NGS data, which are stored as flat FASTQ files on the file system, with corresponding file paths stored in a PostgreSQL database. Considering the sensitivity of participant parameters stored within HIVseqDB, it is essential to underscore that HIVseqDB is specifically deployed on platforms that meet stringent security standards to ensure robust protection of participants’ data integrity.

### 2.4 HIV-1 drug resistance analysis

**Figure 3.**
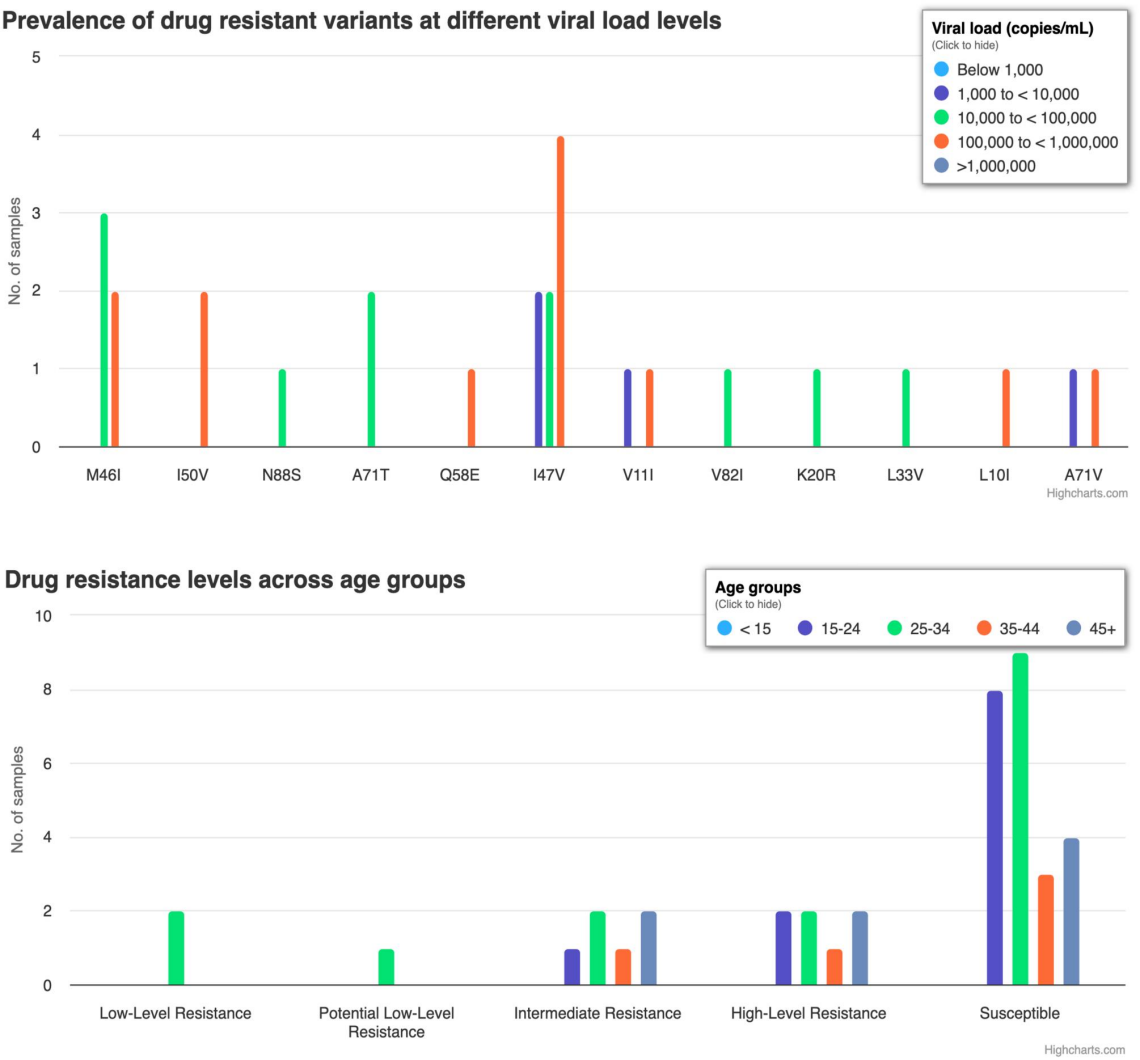
Drug resistance plots. (top) Bar plots showing the prevalence of drug resistant variants of protease inhibitors at different viral load levels. The height of the bars indicates the number of samples in which a particular variant was detected. Bars are colored by the viral load levels. (bottom) Drug resistance to reverse transcriptase inhibitors. The height of the bars indicates the number of samples at a certain susceptibility level. Bars are colored by age group.

The platform provides a page for creating a new analysis, which requires a user to specify the data to be analyzed, and parameter values for the different stages of the analysis. The NGS data are passed to quasitools for quality control, alignment to reference genome, HIV variant calling, and generating consensus sequences. Sierralocal, a local implementation of the Stanford HIVdb database algorithm is used for inferring drug resistance mutations based on the consensus sequences. JSON files generated by sierralocal are processed in R programming interface using R package jsonlite. We use PYTHON libraries JSON, NumPy, and Pandas for numerical computations and data manipulation. Django HTML templates are used to generate drug resistance reports. The drug resistance report shows the susceptibility of a given sample to a particular antiretroviral drug as shown in Supplementary Fig. S1. In addition, HIVseqDB generates a comprehensive report showing all identified variants at mutation frequencies of 1%–100% of the viral population. Data Tables library is used to create interactive tables with pagination and data export functions. Highcharts, a JavaScript library is used to generate interactive visualizations. These plots show mutation frequency and mutational load for drug resistance variants identified in the protease, reverse transcriptase, and integrase regions, the prevalence of resistant variants at different viral load ranges, and drug resistance levels across drug classes for different age groups (Fig. 3).

## 3 Results

We built a portable, effective, and efficient resource for management of HIV-1 NGS data along with associated sample data for analysis of HIV drug resistance. HIVseqDB can be used to provide insights into the relationship between LA-DRMS and clinical/demographic factors. The resource is powered by open-source tools endowed with layers of sequence analysis and result visualization capabilities.

### 3.1 Installation of HIVseqDB

The platform is distributed along with a Docker-compose file that enables smooth installation and deployment across computing environments that support Docker; including Windows, Linux, and MacOS platforms. Installation guidelines are available at https://alfredug.github.io/HIVseqDB/.

### 3.2 Real world utility of HIVseqDB

To demonstrate the utility of HIVseqDB, we obtained a publicly available dataset from the European Nucleotide Archive, Bioproject accession PRJNA340290 and sample metadata obtained from a corresponding publication (Avila-Ríos *et al.* 2016). The dataset comprised paired-end sequence data from 24 samples, with an average of 141 579 reads per FASTQ file.

### 3.3 Runtime analysis

Analysis was performed on a standalone 64-bit workstation with 16 GB RAM and an Intel Core i7, 2.3 GHz processor. Using a network with average internet speed of 131 Mbps, Docker-compose took an average of 17 min to install HIVseqDB and its dependencies. For the dataset mentioned in 3.2 above, it required an average of 4 min per sample to upload, analyze, and generate analysis results.

## 4 Conclusion

HIVseqDB resource provides a portable, secure, and user-friendly platform with low computational resource requirements for integrating NGS data with clinical and demographic data to analyze HIV drug resistance. It is scalable and can be adapted for other viruses with NGS data. The resource employs Docker for portability, making it easy to deploy on a variety of computing environments, including on-premises high-performance computing platforms and cloud-based services.

## Supplementary Material

vbae008_Supplementary_Data

## Data Availability

The NGS data used in this article are publicly available in the NCBI Sequence Read Archive (SRA) and the European Nucleotide Archive (ENA), Bioproject accession number PRJNA340290. Sample metadata are available as part of supporting information for the associated publication (Avila-Ríos *et al.* 2016).
