# Supplementary material for: HIVseqDB: a portable resource for NGS and sample metadata integration for HIV-1 drug resistance analysis
Source: Bioinform Adv. 2024 Jan 14;4(1):vbae008. doi: 10.1093/bioadv/vbae008 (PMC10834361; doi:10.1093/bioadv/vbae008)
Supplement: vbae008_Supplementary_Data [file vbae008_supplementary_data.pdf]

UNHRO
Uganda National Health Research Organisation

Uganda Virus Research Institute
Plot 51-59, Nakiwogo Road, Entebbe
P.O. Box 49, Entebbe-Uganda
Tel: +256 414 320 385 / 6
Fax: +256 414 320 483
Email: directoruvri@uvri.go.ug

REPUBLIC OF UGANDA

HIV Drug Resistance Report

Basic information

Sequence name: SRR4089896_1_SRR4089896_2_1_hxb2_pol

Subtype: B

HIVdb algorithm version: 8.8(2019-02-13)

Gene ranges:

Gene name	First amino acid	Last amino acid
PR	1	99
RT	1	560
IN	1	179

PR: Protease, RT: Reverse Transcriptase, IN: Integrase

Drug resistance interpretation: PR

Major Mutations: None

Minor Mutations: None

Drug Name	Susceptibility	Score
ATV/r	Susceptible	0.0
DRV/r	Susceptible	0.0
FPV/r	Susceptible	0.0
IDV/r	Susceptible	0.0
LPV/r	Susceptible	0.0

Drug Name	Susceptibility	Score
NFV	Susceptible	0.0
SQV/r	Susceptible	0.0
TPV/r	Susceptible	0.0

Drug resistance interpretation: RT

NRTI Mutations: None

NNRTI Mutations: E138A

Drug resistance mutation scoring: NRTI

Drug Name	Susceptibility	Score
ABC	Susceptible	0.0
AZT	Susceptible	0.0
D4T	Susceptible	0.0
DDI	Susceptible	0.0
FTC	Susceptible	0.0
3TC	Susceptible	0.0
TDF	Susceptible	0.0

Drug resistance mutation scoring: NNRTI

Drug Name	Susceptibility	Score
DOR	Susceptible	0.0
EFV	Susceptible	0.0
ETR	Potential Low-Level Resistance	10.0
NVP	Susceptible	0.0
RPV	Low-Level Resistance	15.0

Drug resistance interpretation: IN

Major Mutations: None

Accessory Mutations: None

Drug Name	Susceptibility	Score
BIC	Susceptible	0.0
DTG	Susceptible	0.0
EVG	Susceptible	0.0
RAL	Susceptible	0.0

Comment(s)

E138A is a common polymorphic accessory mutation weakly selected in patients receiving ETR and RPV. It reduces ETR and RPV susceptibility ~2-fold. It has a weight of 1.5 in the Tibotec ETR genotypic susceptibility score.

References

Ho, Jasper C, Garway T Ng, Mathias Renaud, et al. 2019. “Sierra-Local: A Lightweight Standalone Application for Drug Resistance Prediction.” Journal of Open Source Software 4 (33): 1186.

Marinier, Eric, Eric Enns, Camy Tran, Matthew Fogel, Cole Peters, Ahmed Kidwai, Hezhao Ji, and Gary Van Domselaar. 2019. “Quasitools: A Collection of Tools for Viral Quasispecies Analysis.” BioRxiv, 733238.
